# *LEA13* and *LEA30* Are Involved in Tolerance to Water Stress and Stomata Density in *Arabidopsis thaliana*

**DOI:** 10.3390/plants10081694

**Published:** 2021-08-18

**Authors:** Abigael López-Cordova, Humberto Ramírez-Medina, Guillermo-Antonio Silva-Martinez, Leopoldo González-Cruz, Aurea Bernardino-Nicanor, Wilson Huanca-Mamani, Víctor Montero-Tavera, Andrea Tovar-Aguilar, Juan-Gabriel Ramírez-Pimentel, Noé-Valentín Durán-Figueroa, Gerardo Acosta-García

**Affiliations:** 1 Departamento de Ingeniería Bioquímica, Tecnológico Nacional de México/IT de Celaya, Antonio García Cubas Pte. #600 esq. Av. Tecnológico, Celaya 38010, Guanajuato, Mexico; abigael_cordova@hotmail.com (A.L.-C.); hramirez@conacyt.mx (H.R.-M.); guillermo.silva@itcelaya.edu.mx (G.-A.S.-M.); leopoldo.gonzalez@itcelaya.edu.mx (L.G.-C.); aurea.bernardino@itcelaya.edu.mx (A.B.-N.); 2 Departamento de Producción Agrícola, Facultad de Ciencias Agronómicas, Universidad de Tarapacá, Arica 1000000, Chile; whuanca@yahoo.com; 3 Biotechnology Department, National Institute for Forestry Agriculture and Livestock Research (INIFAP), Celaya 38110, Guanajuato, Mexico; montero.victor@inifap.gob.mx; 4 Instituto Politécnico Nacional, Unidad Profesional Interdisciplinaria de Biotecnología, Av. Acueducto S/N., Col. Barrio La Laguna Ticomán, México City 07340, Mexico; andrea.tovar.aguilar@gmail.com (A.T.-A.); nduranf@ipn.mx (N.-V.D.-F.); 5 Tecnológico Nacional de México/I.T. Roque, km 8 Carretera Celaya-Juventino Rosas, Celaya 38110, Guanajuato, Mexico; garamirez@itroque.edu.mx

**Keywords:** LEA proteins, stomata density, drought, plant development, stomata development

## Abstract

Late embryogenesis abundant (LEA) proteins are a large protein family that mainly function in protecting cells from abiotic stress, but these proteins are also involved in regulating plant growth and development. In this study, we performed a functional analysis of *LEA13* and *LEA30* from *Arabidopsis thaliana*. The results showed that the expression of both genes increased when plants were subjected to drought-stressed conditions. The insertional lines *lea13* and *lea30* were identified for each gene, and both had a T-DNA element in the regulatory region, which caused the genes to be downregulated. Moreover, *lea13* and *lea30* were more sensitive to drought stress due to their higher transpiration and stomatal spacing. Microarray analysis of the *lea13* background showed that genes involved in hormone signaling, stomatal development, and abiotic stress responses were misregulated. Our results showed that LEA proteins are involved in drought tolerance and participate in stomatal density.

## 1. Introduction

Abiotic stresses, such as drought, high salinity, heat, cold and freezing, are severe environmental stresses that impair productivity and quality in crop systems and cause extensive losses to agricultural production worldwide because they affect both vegetative and reproductive plant development. However, drought and heat stress usually significantly impact plant reproduction [1,2].

As sessile organisms, plants have developed various sophisticated strategies for adapting to unfavorable environmental changes, including signal transduction to trigger the activation of specific stress-related genes [1,3,4,5].

One of the response mechanisms used by plants during drought stress is the control of stomata closure to reduce transpiration and, therefore, water loss. During stomata development, there are at least three transcription factors that determine the cell type to be differentiated, which are SPEECHLESS (SPCH), MUTE, and FAMA. SPCH regulates the meristemoid mother cell (MMC) transition and asymmetric cell divisions. MUTE regulates meristemoid transition in the guard mother cell (GMC), while FAMA regulates asymmetric divisions that give rise to guard cells (GC) [6]. Additionally, stomatal density and pattern are determined by signals involving the EPIDERMAL PATTERNING FACTORS (EPFs) family, plant hormones, and environmental clues. Because SPCH is early in stomatal development, most act by activating or repressing SPCH, these signals include leucine-rich repeat receptor kinases (LRR-RKs), Cysteine-Rich Receptor-Like Kinases (CRKs), ERECTA (ER), ER-LIKE 1 (ERL1), ERL2, and TOO MANY MOUTHS (TMM) an LRR receptor protein [7,8].

Moreover, late embryogenesis abundant proteins (LEA proteins) are a group of proteins that were first identified in cottonseed (*Gossypium hirsutum*) and wheat (*Triticum aestivum*) during the late stages of embryogenesis [1]. They also have been found in other organs in different plants, especially under stress conditions such as cold, high salinity, and drought [5,9,10]. 

Tobacco plants overexpressing *CaLEA6* (from *Capsicum annuum*) show enhanced tolerance to dehydration and NaCl but not to colder conditions, as defined by their fresh leaf weights. Therefore, the *CaLEA6* protein plays a potential protective role when a water deficit is induced by dehydration and high salinity but not by low temperature [3]. *MsLEA4-4* from *Medicago sativa* was shown to be involved in drought, salt, and oxidative tolerance when it was overexpressed in Arabidopsis plants [11]. *MtCAS31* from *Medicago trunculata* enhances drought tolerance in transgenic Arabidopsis by reducing stomatal density. 

From *C. annuum* cv. caballero seeds using differential expression approaches, an *LEA* gene (*CaLEA73*) was identified, and its complete cDNA was isolated. *CaLEA73* is highly induced in osmopriming treatments, in which KNO_3_ instead of GA_3_ was used in combination with PEG [12]. The *CaLEA73* gene was ectopically expressed in transgenic *A. thaliana* to analyze its role under drought and salt stresses. The results showed an increase only in drought tolerance in the transgenic lines evaluated [13].

The molecular mechanisms activated when *CaLEA73* is overexpressed and the endogenous genes that perform a similar function in *Arabidopsis* remain unknown. The present study identified two genes, namely At2g18340 and At3g17520, as related to *CaLEA73*. 

Previous reports have suggested the nomenclature for LEA genes. Bies-Ethève et al. [14] named these genes according to how they were placed in different groups and named At2g18340 as *AtLEA3-14* and At3g17520 as *AtLEA4-3*. In total, 51 genes have been found in the *Arabidopsis* genome that encodes LEA proteins, also named with a number according to their position in the *Arabidopsis* genome, starting at the top of chromosome 1 [15,16]. Following the above, we named the genes of interest *LEA13* (At2g18340) and *LEA30* (At3g17520).

Previous reports have shown that *LEA13* and *LEA30* are involved in the salt stress response and could be in the ABA pathway [14,16,17]. A very comprehensive study on the cellular localization of LEA proteins in Arabidopsis found that both *LEA13* and *LEA30* colocalize in the endoplasmic reticulum and, interestingly, were also observed in vacuoles and the plasma membrane, suggesting they collaborate in similar functions. 

Our results indicated that *LEA13* and *LEA30* are involved in responding to water stress as they are upregulated in wild-type plants under stress conditions. Additionally, *LEA13* and *LEA30* are required for a proper stomatal density. Physiological studies showed a high transpiration rate, photosynthesis rate, and stomatal conductance in *lea13* and *lea30* compared to wild-type plants, which caused the loss of water. Stomata development was also affected in *lea13* and *lea30* lines, as the expression of stomata cell fate markers was affected. 

## 2. Results

### 2.1. Identification of Genes Related to CaLEA73

**Figure 1:**
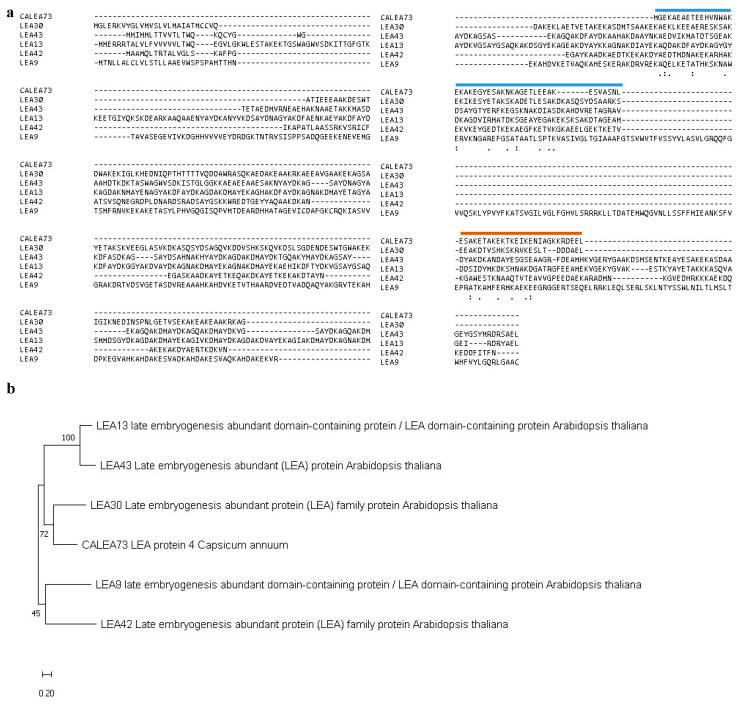
The alignment of the amino acid sequences and phylogenetic relationships between selected proteins and CaLEA73. (**a**) Amino acid sequences alignment of candidate proteins. They were aligned using the ClustalW program; dashed lines were introduced to maximize sequence alignments. The blue line indicates the first conserved region and the orange line second conserved region. (**b**).

A search of the Arabidopsis database using the BLAST algorithm was performed to identify sequences related to *CaLEA73* and select *LEA* genes for functional analysis. The analysis allowed the identification of LEA13 and LEA30. Three additional amino acid sequences of the proteins LEA9, LEA42 and LEA43 were compared with CaLEA73 because they had also been reported to be related to LEA13 and LEA30 [15] to increase the robustness of the analysis. Alignment analysis was performed (Figure 1a) to analyze the existence of conserved domains possibly related to their function. The alignment analysis revealed that CaLEA73 and LEA13 shared 40.0% identity and 66.0% similarity, CaLEA73 and LEA30 shared 45.7% identity and 75.7% similarity and LEA13 and LEA30 shared 36.2% identity and 58.7% similarity. Two conserved regions of 37 and 27 amino acids were observed between the sequences, which may be involved in the function of water stress tolerance or stomata development. 

A phylogenetic tree was generated to obtain a better idea of how these LEA genes might be related between them (Figure 1b). The result showed that in the same clade of CaLEA73 are LEA13, LEA30, and LEA43, suggesting they may have a similar developmental function.

The phylogenetic tree of amino acid sequences of selected proteins was created. MEGA X software was employed to generate the relationships between five Arabidopsis LEA proteins and the *Capsicum annuum* protein CaLEA73. The percentage of trees in which the associated taxa clustered together is shown next to the branches.

A comparison of the shared regulatory elements in *LEA13* and *LEA30* by in silico study was performed to understand the regulatory mechanisms of LEA genes. The 0.5-kb sequences upstream of the translation start site were used as promoters of the *LEA13* and *LEA30* genes; these were obtained from the TAIR’s SeqViewer. The transcriptional response elements in the LEA genes promoters were analyzed using the PLACE database. To determine cis-acting regulatory elements, we queried the sequence of each promoter, analyzed, and identified the presence of various stress-responsive cis-acting regulatory elements, including DRE/CRT, ABRE, LTRE, and MYBS. These stress-responsive elements were relatively abundant in both promoters of the LEA genes, specifically ABRE for *LEA13* and DRE for *LEA30* (Figure S1). These in silico findings suggested responsiveness to abiotic stress for both LEA genes. 

### 2.2. LEA13 and LEA30 Are Involved in the Tolerance to Water Deficit Stress

Insertional lines were identified for each gene, *lea13* (SALK_004249) and *lea30* (SALK_077676), from the Salk Institute database to analyze the function of the *LEA13* and *LEA30* genes during plant development. The analysis showed that these genes have a T-DNA insertion in the regulatory region (Figure S2). 

**Figure 2:**
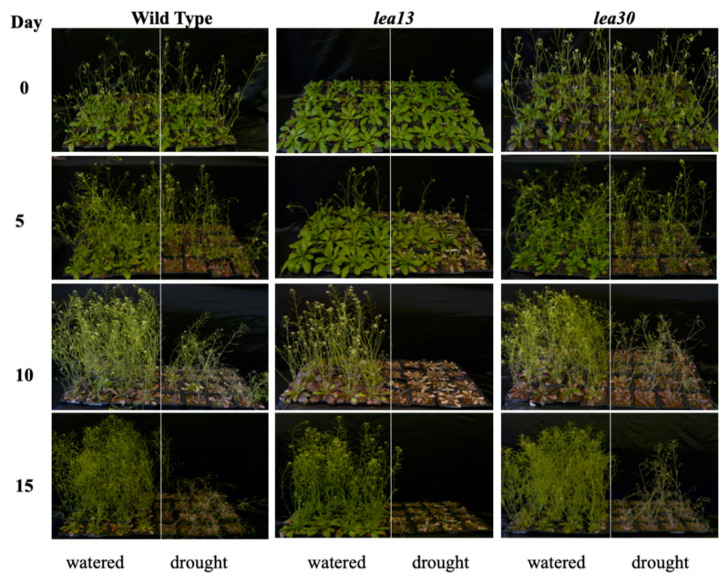
Tolerance to water deficit stress. Six-week-old plants were subjected to water stress, sampled at days 0, 5, 10, and 15 after cessation of irrigation, and analyzed for overall plant phenotype. The *lea13* and *lea30* lines showed greater susceptibility to water stress than the wild-type, which was more evident on day 10. Although all plants were planted and grown in the same space and conditions, the *lea13* and *lea30* lines showed a delay in development regardless of whether they received irrigation or drought, more pronounced in the *lea13* line. Ten plants for each line were used as the experimental unit. Photographs were taken just in the drought stress (Left control plants were well-watered, and on the Right side are plants subjected to drought stress).

A water content (WC) analysis was performed to determine how water content changes in *lea13* and *lea30* mutant plants compared to the wild-type line. For this, insertional lines and wild-type plants were submitted to a drought test for 15 days (Figure S3). During the initial 5 days after withholding water, there was no significant decrease in the amount of plant water neither in the wild-type lines nor *lea13* and *lea30*. Ten days after cessation of irrigation, however, the differences became evident as it was relatively higher in the wild-type (90%) than in *lea13* and *lea30* plants (less than 80%). *lea13* plants lost a higher water content than *lea30* (Figure S3). When plants grown on soil were subjected to drought stress (15 d of water deprivation), a clear difference in phenotype was observed between *lea13*, *lea30*, and wild-type plants (Figure 2). Wild-type plants continued to grow, albeit at a slower rate, whereas *lea13* and *lea30* exhibited chlorosis and died. Although *lea13* plants were more sensitive to drought stress conditions than *lea30* plants in rosette leaves, both showed a remarkable delay in growth (Figure 2). In general, *lea13* plants showed a more robust phenotype than *lea30*, and according to phenotypic analysis, *LEA13* and *LEA30* are implicated in general plant physiology and development. 

### 2.3. Expression and Functional Analyses of LEA13 and LEA30 under Water Stress Conditions

**Figure 3:**
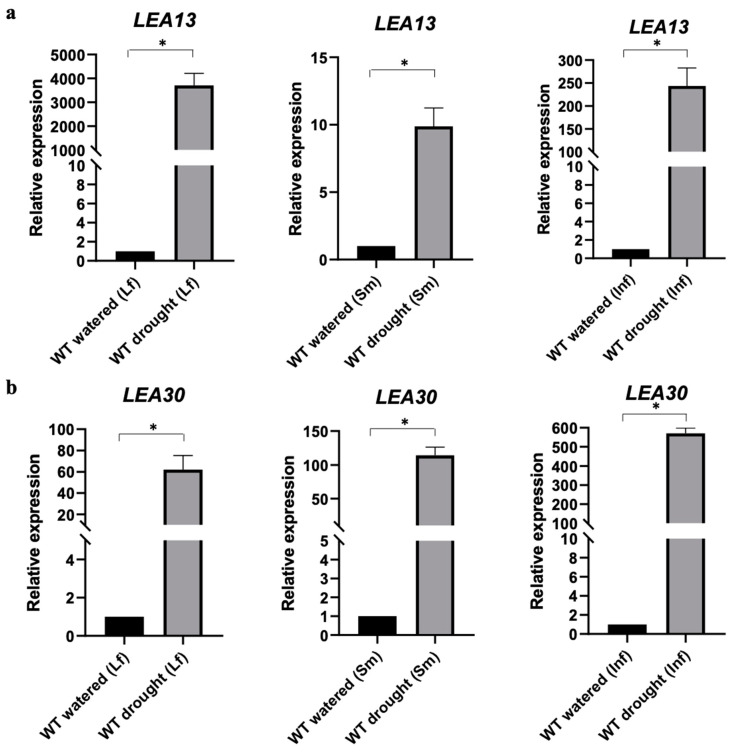
Quantitative RT-PCR analysis of *LEA13* (**a**) and *LEA30* (**b**) on water stress conditions. Wild-type plants of *Arabidopsis thaliana* were left without watering for ten days, then RNA samples were taken from rosette leaves. Abbreviations: WT (wild-type), Lf (rosette leaf), Sm (stem), and Inf (inflorescence). Y-axis: relative expression (2^−ΔΔCT^). * Data represent samples significantly different *p* < 0.05. All tests were performed using 3 biological and technical replicates.

To determine the expression of *LEA13* and *LEA30* under water stress conditions, 3-wk-old plants with no further water addition for 10 d (drought). The tissue subjected to stress and the control (wild-type plants watered daily) was obtained to perform a quantitative PCR assay using stem, inflorescence, and rosette leaf tissues. We found that the two genes had different patterns of transcript accumulation during tolerance. The relative expression (2^−ΔΔCT^) of *LEA13* in wild-type plants increased when plants were subjected to drought stress up to 3600-fold for leaf, 9-fold for the stem, and 225-fold for inflorescence. *LEA30* also showed an increase during drought to 60-fold for leaf, 110-fold for the stem, and 550-fold for inflorescence (Figure 3). An in silico analysis using 20,000 publicly available RNA-seq libraries [18] for *LEA13* and *LEA30* was performed (Figure S4). This analysis confirmed that *LEA13* and *LEA30* are expressed during seed development and lowly in leaf, stem, and flower (Figures S4A and S4B). Interestingly, *LEA13* and *LEA30* expression is induced upon stress conditions, mostly during drought (Figures S4C and S4D). 

**Figure 4:**
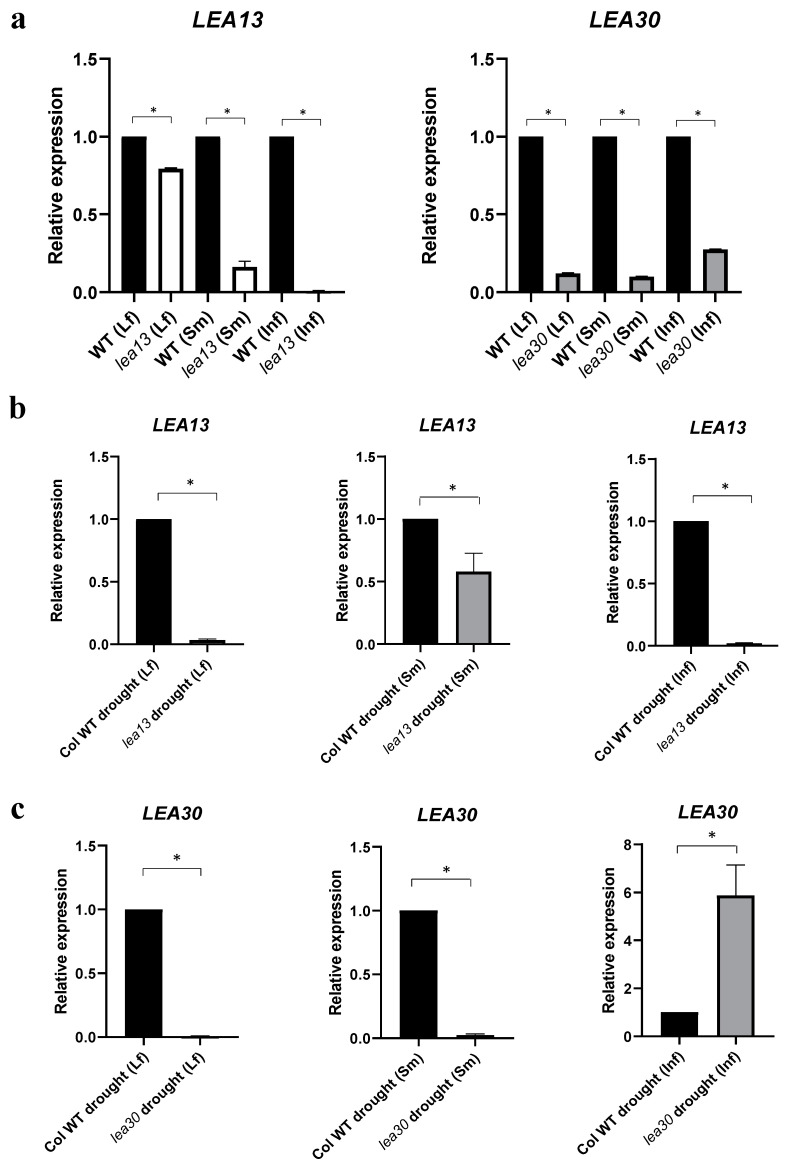
The expression pattern of *LEA13* and *LEA30* genes in *lea13* and *lea30* during tolerance to water stress. (**a**) Relative expression of *LEA13* in background *lea13* and relative expression of *LEA30* in background *lea30*. (**b**) Relative expression of *LEA13* in background *lea13* during tolerance to water stress. (**c**) The relative expression of *LEA30* in background *lea30* under water stress conditions. Abbreviations: Sm (stem), Inf (inflorescence), and Lf (rosette leaf). After 10 days of water deficit. Y-axis: relative expression (2^−ΔΔCT^). * Data represent samples significantly different *p* < 0.05. All tests were performed using 3 biological and technical replicates.

A qRT-PCR expression analysis was performed to determine the *LEA13* and *LEA30* expression in mutant genetic backgrounds. The relative expression *LEA13* in the *lea13* genetic background decreases significantly for leaf, stem, and inflorescence. The relative expression of *LEA30* in the *lea30* background also decreases significantly for leaf, stem, and inflorescence (Figure 4a). During drought (ten days after the plants stopped being irrigated), *LEA13* expression decreased in *lea13* background compared to wild-type (Figure 4b). *LEA30* showed similar behavior; in leaf and stem, the expression in the mutant background decreased compared to the wild-type, while in inflorescences tissue, the expression increased (Figure 4c). 

### 2.4. Transpiration and Stomatal Density Are Higher in lea13 and lea30

**Figure 5:**
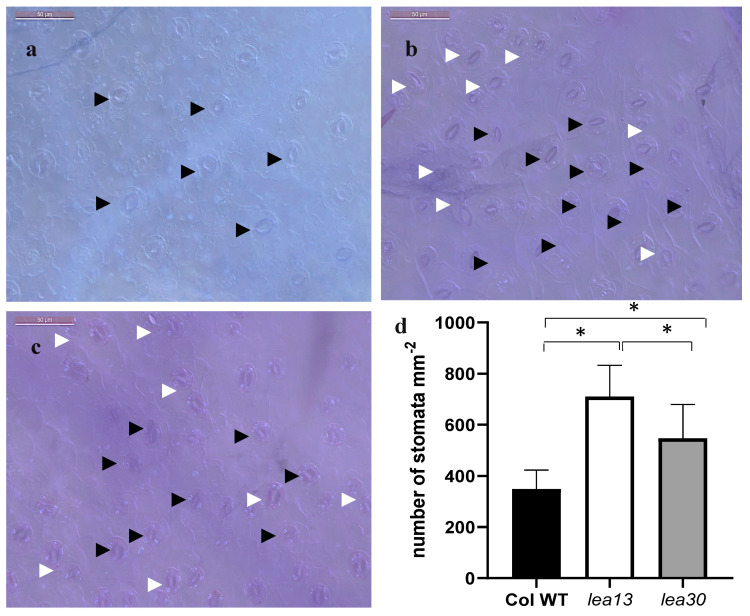
Stomatal density in *lea13*, *lea30*, and wild-type plants (**a**–**c**). Adaxial sides of cotyledons. Bar, 50 μm. (**a**) Wild-type. (**b**) *lea13*. (**c**) *lea30*. (**d**) Stomatal density is shown as the average number of stomata per square millimeter. Black arrowheads point mature stomata. White arrowheads point stomata clusters. Bars indicate standard deviation. * Data represent samples Col WT and *lea13* significantly different *p* < 0.05. ** Data represent samples Col WT and *lea30* significantly different *p* < 0.05.

**Figure 6:**
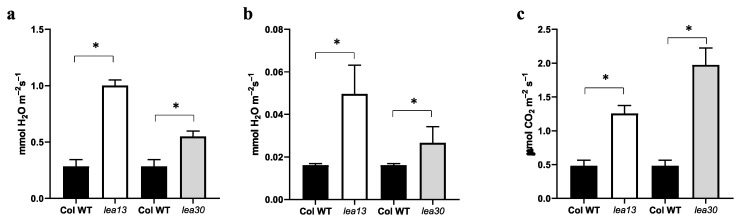
Transpiration, stomatal conductance, and photosynthesis. Rosette leaves of 10 plants of each line were analyzed. (**a**) Transpiration, (**b**) Stomatal conductance, and (**c**) Photosynthesis levels in *lea13*, *lea30*, and wild-type plants. The measurements were performed at a temperature of 25 °C and vapor pressure between 1 and 1.3 kPa with a relative humidity of 70%. Bars indicate standard deviation. * Data represent samples significantly different *p* < 0.05.

Phenotype analysis of cotyledons showed an increase in stomatal density in the mutant lines *lea13* and *lea30* compared to wild-type (Col-0). Observations on the epidermal surface of cotyledons in the wild-type background showed that stomata are at the mature stage and with a pavement cell separating them (Figure 5a). In the *lea13* and *lea30* mutants, stomata were also observed at the mature stage; however, the spacing varied (Figure 5b,c). In some areas, clusters of stomata were observed without spacing cells. This phenotype was observed to be stronger in the *lea13* background, and the stomata number was higher than in *lea30* (Figure 5d). Physiological parameters were measured to analyze the functionality of the stomata. The transpiration rate and stomatal conductance were higher in *lea13* and *lea30* than in wild-type plants (Figure 6a–b), resulting in increased photosynthesis in *lea13* and *lea30* compared to that in wild-type plants (Figure 6c). These results suggested that *LEA13* and *LEA30* are involved in the determination of the stomatal patterning and density.

### 2.5. Stomata Developmental Genes Expression Is Affected in the 35S:CaLEA73 Background

**Figure 7:**
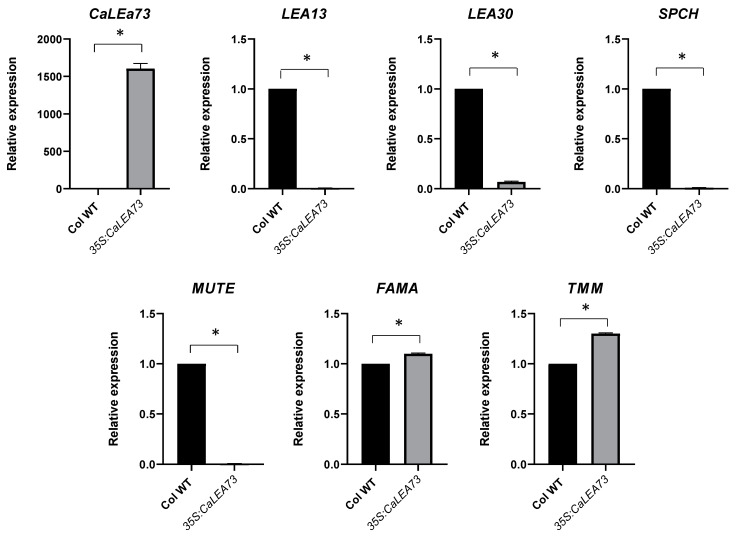
Analysis of the gene expression of *lea13*, *lea30* and markers of stomata development in the *35S:LEA73* genetic background. Cotyledons of seedlings 15 days after germination (dpg) growth under normal irrigation conditions were analyzed. Y-axis: relative expression (2^−ΔΔCT^). * Data represent samples significantly different *p* < 0.05. All tests were performed using 3 biological and technical replicates.

Arabidopsis plants overexpressing the *CaLEA73* gene from the *Capsicum annuum* showed a phenotype of lower stomatal density and higher tolerance to hydric stress than wild-type plants [13]; this phenotype is the opposite to that observed in *lea13* and *lea30*. To determine whether stomata development is affected when LEA protein levels are altered, the expression of cell fate marker genes *SPCH*, *MUTE*, *FAMA*, and *TMM*, in the stomata of rosette leaves in 35S:*CaLEA73* was analyzed (Figure 7). The expression of *LEA13* and *LEA30* in 35S:*CaLEA73* rosette leaves was repressed compared to wild-type. The expression pattern of *SPCH* and *MUTE* decreased significantly, but the expression of *FAMA* and *TMM* increased significantly in 35S:*CaLEA73* (Figure 7). These results suggest that the formation of new stomata has been stopped and that the misregulation of *LEA* genes alters stomata development.

### 2.6. Differential Gene Expression in lea13

**Figure 8:**
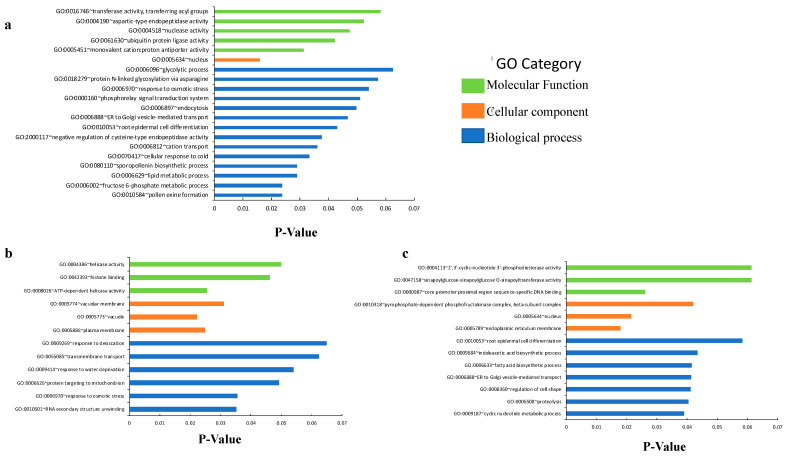
Significantly enriched Gene Ontology (GO) categories (biological process, cellular component, and molecular function): (**a**) associated with differentially expressed genes from *lea13* microarray data, (**b**) associated with upregulated genes from *lea13* microarray data, and (**c**) associated with downregulated genes from *lea13* microarray data. Fisher Exact *p*-Value = 0 represents perfect enrichment. Usually, *p*-Value is equal to or smaller than 0.05 to be considered strongly enriched in the annotation categories.

Because the *lea13* line showed a stronger and more consistent phenotype on stomatal density and patterning, we decided to analyze the transcriptional activity in this genetic background in plants grown under normal irrigation conditions, where *LEA13* expression is reduced compared to the wild-type background. Microarray analysis showed that 1201 genes were altered more than twofold in the *lea13* genetic background (Figure 8), with 589 upregulated genes (Table S1) and 612 downregulated genes (Table S2). Functional gene analysis of misregulated genes showed an enrichment within Gene Ontology (GO) terms corresponding to biological processes such as response to desiccation, response to water deprivation, or response to osmotic stress. However, it also highlights genes related to signaling processes, membrane modification, cell transport, and cell differentiation processes (Figure 8a). In the case of upregulated genes, genes related to drought and osmotic tolerance, transmembrane transport in the cells and organelles are highlighted (Figure 8b). Down-regulated genes were more focused on differentiation processes, cell signaling, hormones, and cell shape and membrane (Figure 8c). 

## 3. Discussion

Low water availability caused by different environmental conditions, such as drought, salinity, or low temperatures, represents a vulnerable situation for many plants and crops. To contend with and overcome these adverse environments, numerous response mechanisms have been developed by different species of the plant kingdom [19]. LEA proteins are involved in the tolerance to drought stress [15,20,21]. On the other hand, there is little information on the participation of LEA proteins in cell differentiation or signaling processes during plant development.

Based on the previously reported *CaLEA73* gene function, *LEA13* and *LEA30* were identified in the present study. Although CaLEA73 is a very small protein (only 73 amino acids), it shares two domains with selected Arabidopsis LEA proteins [16,20,22,23,24]. The first domain with 39 amino acids and the second domain with 27 aa, and the region shared among the 5 selected genes (Figure 1). The above suggests that these conserved regions may be involved in drought tolerance and the regulation of stomatal density. In addition, a phylogenetic tree from the amino acid sequences of the candidate proteins established a closer relationship between CaLEA73, LEA13, LEA30, and LEA43. Analysis of the promoter region of *LEA13* and *LEA30* allowed the identification of stress-responsive cis-acting regulatory elements, mostly ABRE, DRE, MYBS, and LTRE (Figure S1). Most of these cis-elements are involved in the ABA-dependent signaling pathway in response to abiotic stresses.

*LEA13* and *LEA30* were found to be involved in the plant response to water stress in leaf, stem, and inflorescence tissues. Both genes showed low expression when the plant was not subjected to stress, but their transcript levels increased when plants were under drought stress conditions (Figure 3). *LEA13* expression increased more in leaves than in stems or inflorescences, while LEA30 increased in inflorescences than in leaves or stems. The *lea13* and *lea30* insertional lines contain a T-DNA in the regulatory region, affecting the ABRE and DRE domains interaction (Figure S1–S2), resulting in downregulation of both genes when plants were under normal irrigation conditions (Figure 4). However, when plants were subjected to stress, *LEA13* and *LEA30* increased their expression in leaves, even in the mutant background (Figure S5), although to a much more limited extent than in the wild-type. The response differed in stem and inflorescence as *LEA13* showed higher expression in both tissues in the mutant background than in the wild-type. In the case of *LEA30* only in inflorescences, the expression was higher in the *lea30* background than in the wild-type. These results suggest that *LEA13* and *LEA30* may have different regulatory mechanisms and perhaps functions in other tissues. One possibility is that transcription factors can still recognize the ABRE and DRE cis-elements in the insertional lines, but they are different, so the response in terms of expression is different in each tissue.

*OsLEA3-1* (from *Oryza sativa*) has been identified and overexpressed in rice to test the drought resistance of transgenic lines under field conditions. *OsLEA3-1* is induced by drought, salt, and abscisic acid (ABA) but not by cold stress, and transgenic rice significantly increases drought resistance [25]. Previous reports have shown that overexpression of *CaLEA6* increases tolerance to drought and NaCl but not too low temperature, suggesting that LEA proteins may be involved in different types of stress tolerance. Dang et al. [26] screened for possible functions of LEA proteins in stress tolerance by expressing 15 genes from *A. thaliana* in *Saccharomyces cerevisiae*, and their desiccation stress experiments showed that eight of the 15 LEA proteins significantly enhanced yeast survival [15,27].

Water content analysis showed that the *lea13* and *lea30* lines lose water content faster than the wild-type, while *lea13* showed a higher loss than in *lea30*; it also was confirmed by phenotyping analysis (Figure 2), confirming that LEA proteins are part of the plant’s response mechanism to drought conditions.

Xu et al. [28] reported that the expression of *HVA1*, an LEA III family protein in barley, confers tolerance to water deficiency in transgenic rice plants. They reported that constitutive expression of the same protein in transgenic wheat plants improves biomass productivity [28]. Salleh et al. [29] found that AtLEA5, in addition to responding to drought stress conditions, overexpressing lines affect flowering time, shoot biomass, and root length. In addition, it has been reported that the differential of expression of some *LEA* genes in different tissues suggesting a function during plant development [30]. 

The integrated response of the whole plant to water deficit must also encompass sensing and signaling mechanisms. The ABA plant hormone is the best-known signal at both the whole plant and cellular levels. ABA can move throughout the plant in the vascular system, and it acts as a signal for changes in stomatal conductance and gene expression in response to soil drying. It is now clear that stress responses are dependent on the tissue, cell type and developmental stage of the plant [1,20].

In this study, the downregulation of *LEA13* and *LEA30* led to increased stomatal density and the transpiration rate, photosynthesis, and stomatal conductance. These latter results suggested an additional function of *LEA13* and *LEA30* in stomatal density. Moreover, stomata failed to close in the *lea13* and *lea30* lines. One of the main hormones that have been reported to be involved in stomata closing is ABA [17,31,32]. One hypothesis is that *LEA13* and *LEA30* are required for the correct function of ABA. Previous reports have analyzed several LEA proteins and have found that *LEA13* and *LEA30* are expressed in most tissues (seed, stem, flower, and leaf) and that LEA30 is induced by ABA treatment [14]. In addition, observations of the adaxial epidermal surface of cotyledons of *lea13* and *lea30* showed defects in stomata development (Figure 5), as most were at the mature stage, but there were stomatal clusters without the cell spacing that normally allows the correct stomatal patterning.

Acosta-García et al. overexpressed the *CaLEA73* gene of *C. annuum* in *A. thaliana*. The transgenic plants increased drought tolerance due to lower transpiration levels and a lower stomatal density than those in control plants [13]. Thus, these overexpressing lines 35S:*CaLEA73* showed a phenotype opposite to that observed in *lea13* and *lea30*, suggesting that high levels of some LEA proteins decrease stomatal density while low levels increase stomatal density. In addition, the *MUTE* and *SPCH* stomata genes were downregulated, which agreed with the phenotype of higher stomatal density in *lea13* and *lea30*. While TMM and FAMA increased their expression (Figure 7), these results suggest that asymmetric divisions stopped and decreased the number of stomata. In the *lea13* and *lea30* lines, where there are low levels of LEA proteins, the phenotype is similar to that of *tmm* (Figure 5). *LEA13* and *LEA30* transcripts were reduced and hardly detectable in *35S:CaLEA73* background, which suggests that if there are high levels of this type of LEA protein, the expression of *LEA13* and *LEA30* is either not required or is downregulated (Figure 5). Nevertheless, low levels of LEA proteins may upregulate the expression of another LEA gene (Figure S6). 

We compared gene expression levels in the *lea13* T-DNA line using an Arabidopsis whole genome microarray to find genes related to LEA protein function. Differentially expressed genes were identified by comparing the expression profiles of *lea13* and *Col-0* cotyledons. These genes are misregulated due to LEA13 downregulation can be grouped into 3 main groups, the first one related to stress response, the second one related to stomata development, and the third one related to general plant development, mainly flowering (Figure 8). Interestingly, many of the differentially expressed genes are related to the endoplasmic reticulum where both LEA13 and LEA30 have been localized and may be involved in protein modification under stress conditions. Additionally, genes coding for proteins with DNA binding capacity were observed, which could be related to stomata or plant development processes.

One of the upregulated genes was SAUR-like auxin-responsive protein is a hormone-related gene (Table 1). By transcriptome analysis, Yang et al. [33] found that SAUR-like is related to xylem differentiation processes and regulating genes involved in cell division processes. Additional experiments will be required to analyze which of these events are involved during stoma development. GSK3/Shaggy kinases are involved in a wide range of fundamental processes in development and metabolism. GSK3/Shaggy kinases have been involved in the response and adaptation to salt stress [34]. Perhaps these proteins are involved in the signaling pathway that LEA proteins activate when plants are subjected to stress conditions.

*SPT6-like* (*SPT6L*) is a conserved elongation factor that is associated with phosphorylated RNA polymerase II (RNAPII) during transcription (Table 1). Chen et al. [35] mentioned two versions of SPT6: SPT6 and SPT6-like (SPT6L) in *Arabidopsis*. The transcript of *SPT6* is poorly detected in most tissues, and mutants do not show an obvious phenotype. SPT6L is involved in determining the apical-basal axis and embryonic development. Therefore, SPT6L may be involved in the asymmetric divisions that occur during stomata development.

Members of the plant-specific gibberellic acid-stimulated *Arabidopsis* (GASA) gene family play roles in hormone responses, defense, and flower development. Gibberellins have been reported to participate in many biological processes, including cell differentiation [36]. Gibberellins are related to stomata formation in *Arabidopsis* hypocotyls and cell division [37], and they participate in the processes of plant transpiration and drought tolerance [22]. EPIDERMAL PATTERNING LIKE (EPFL) encodes peptides involved in plant development, which decrease stomatal density when overexpressed [38,39]. These findings match the stomatal density observed in both *lea13* and *lea30*.

Another gene that has attracted attention is the *FLOWERING LOCUS T* (*FT*) gene, which has been shown to modulate ambient temperature-responsive flowering in *Arabidopsis* [40]. An interesting phenotype observed in *lea13* and *lea30* was a delay in the development and flowering of the plant. This delay may have been due to the expression of the *FT* signaling pathway being affected. However, to confirm this, additional experiments are required. 

Transcription factors were also found in the downregulated genes (Table 2), including *NAC9*, *AINTEGUMENTA-like 7*, *MYB*, *bHLH123*, and *AGAMOUS-like 93*, which participate in different developmental programs, such as floral development, cell division, lateral root development, senescence, secondary cell wall synthesis, abiotic stress responses, and biotic stress responses [41,42,43,44]. In Arabidopsis, FLP1 and MYB88 encode two paralogous R2R3-MYB proteins, which involves establishing stomatal patterning by permitting only a single symmetric division before stomata differentiate and in the control of abiotic stress responses [45]. Double mutant flp1 myb88 produce plants with abnormal stomata development are more susceptible to drought and salinity stress and have increased water loss than WT plants [45,46]. Similar phenotypes are described to *lea13* and *lea30*. When overexpressed, these transcription factors reveal their relationship with the phytohormone auxin, auxin is a plant hormone that widely regulates plant development, but its role in stomatal development was reported only recently. An interesting time-lapse experiment reveals that auxin activity changes over stomatal development. Auxin activity is high in the early stages but depleted from GMCs [8]. This result could explain the growth and flowering delay in the lines *lea13* and *lea30*.

Wurzinger et al. [47] mentioned that *AtMPK9* (At3g18040) and *AtMPK12*, which are preferentially and highly expressed in guard cells, also function as positive regulators of stomatal closure. The CLAVATA3/ESR (CLE)-related genes have been suggested to regulate meristem maintenance and the undifferentiated state [48,49]. A possible explanation for the increased transpiration in the *lea13* and *lea30* lines is that the closure of the stomata is inactive, as these could be involved in the processes of stomata differentiation.

**Figure 9:**
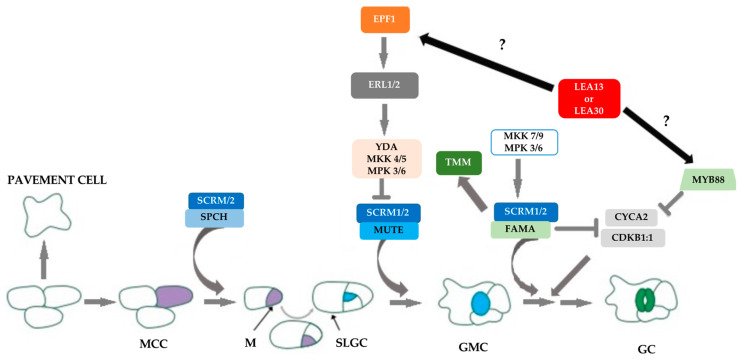
The hypothetical role of LEA proteins during stomatal development in Arabidopsis. A part of the protodermal cells acquires the character of meristemoid mother cells (MMCs); the MMC undergoes asymmetric cell division and gives rise to a meristemoid (M) and a stomatal lineage ground cell (SLGC). Meristemoids can undergo asymmetric amplifying divisions before differentiating into a guard mother cell (GMC). GMC undergoes symmetric division to form a pair of guard cells (GC), forming SCRM1 or SCRM2, playing essential roles in the respective key steps, as indicated by arrows. FLP1 and MYB88 are MYB transcription factors that regulate key fate transitions during stomatal development. *LEA13* and *LEA30* can both activate directly or indirectly on FLP1/MYB88 and some cyclins for modulation of cell divisions or EPF1, a peptide secreted from meristemoids and GMC cells, is perceived by a receptor complex consisting of ERL1, TMM, and SERKs, which prevents the meristemoid from producing stomata next to the existing stoma.

Kanaoka et al. [50] reported that the ICE1 protein forms a dimer with these transcription factors to regulate stomatal development. Genes related to helix-base (bHLH), namely, *SPCH*, *MUTE*, and *FAMA*, are positive regulators of entry into the stomatal cell linage, transition from the meristem to CMG, and terminal differentiation of guard cells, respectively [23,24,50,51,52,53,54]. These expression changes suggest that altered *LEA13* and *LEA30* expression affect stomatal density and development by modifying the expression of key genes for stomata development. In the case of the role of LEA proteins in determining stomatal density, based on the results obtained in this study and previously reported, we hypothesized (Figure 9) that under certain stress conditions, LEA13 and LEA30 can activate directly or indirectly on MYB proteins such as FLP1 or MYB88 and some cyclins for modulation of cell divisions. Another possibility is that low levels of LEA proteins also activate EPF1, a peptide secreted from later stomatal lineage cells (late meristemoids and GMCs), which is perceived by a receptor complex consisting of ERL1, TMM, and SERKs in neighboring meristemoids, which prevents the meristemoid from producing stomata next to the existing stomata. In both cases, in *lea13* or *lea30* mutant, the interaction with these proteins is altered, giving rise to defects in stomatal development and activating a mechanism to increase stomatal density [39,55,56].

In addition, LEA protein levels may influence the expression of other LEA genes and plant developmental genes, allowing plants to have a faster response, which includes activation of water stress response genes, plant development, and stomatal density. 

## 4. Materials and Methods 

### 4.1. Plant Materials and Growth Conditions

T-DNA insertion alleles for *LEA13* (SALK_004249; *lea13*) and *LEA30* (SALK_077676; *lea30*) were obtained from the *Arabidopsis* Biological Resources Center (ABRC) at Ohio State. *Arabidopsis thaliana* plants ecotype Columbia-0 (Col-0) was used as wild-type in all studies unless otherwise noted. The T-DNA lines were confirmed by PCR-based genotyping using primers designed by primer BLAST. The following primers were used: *LEA13-F* (5′-TTTATCACCAGAGAATCAGAC-3′); *LEA13-R* (5′-TTTCCTCTTGCCACGTCAACA-3′); *LEA30-F* (5′-TTGGACACAACACATCGT-3′); and *LEA30-R* (5′-GAAGATCGGAAATCATCA-3′). Plants were initially grown on Murashige and Skoog (MS) agar plates in a plant growth chamber (Lab-line biotronette-model) with 16 h light/8 h dark cycles for 10 days at 23 °C. Then, seedlings were transferred to pots containing substrate (peat moss: perlite: vermiculite 3:1:1 v/v) in a greenhouse with natural light (PAR of 140 µmol m^−2^ s^−1^) at 25 °C with an average maximum and minimum relative humidity of 80% and 60%, respectively.

#### Sequence Alignments

The three LEA proteins, namely, LEA13 (At2g18340), LEA30 (At3g17520), and CaLEA73, were selected for amino acid sequence alignment by Clustal Omega software (https://www.ebi.ac.uk/Tools/msa/clustalo/). The phylogenetic tree was inferred by using the Maximum Likelihood method and General Reverse Transcriptase + Freq. model [57]. The tree with the highest log likelihood (−4625.43), based on 100 bootstraps, is shown. This analysis involved 6 amino acid sequences. All these analyses were conducted in MEGA X [58].

### 4.2. Promoter cis-Element Analysis

The promoter sequences (0.5 kb upstream of the translation start site) of all LEA genes were obtained from The *Arabidopsis* Information Resource (TAIR) (https://www.arabidopsis.org/). The transcriptional response elements in the LEA gene promoters were predicted using the PLACE database (https://www.hsls.pitt.edu/obrc/index.php?page=URL1100876009).

### 4.3. Drought Tolerance Experiments and Determination of Water Content

Seeds of wild-type (Col-0 ecotype), *lea13*, and *lea30* lines were grown as mentioned above. Three-week-old plants were transferred to a chamber at 23 °C with no further water addition for 15 d (drought) to determine the expression of LEA13 and LEA30 genes under water stress conditions. Control wild-type and mutant plants were watered daily. The tissue was obtained from 10 drought-stressed plants and 10 normally irrigated plants on days 0 and 15. Water Content (WC) was determined as follows: 3 rosette leaves of each plant by treatment were excised, and their fresh weight was scored immediately. Leaves were then dried in an oven at 70 °C overnight and weighed. The Water Content was calculated as follows (Equation (1)):

(1) $$WC=\frac{\left(freshweight-dryweight\right)}{freshweight}\cdot 100$$

### 4.4. Gene Expression Analysis by qRT-PCR

To test the expression levels of *LEA13* (At2g18340), *LEA30* (At3g17520), and *AtUBC10* (AT5G53300), an internal control [59,60] using qRT-PCR, total RNA was extracted using TRIzol reagent according to the manufacturer’s instructions (Invitrogen, MX). The RNA concentration was checked using a NanoDrop 2000 spectrophotometer. Approximately 500 ng of total RNA was used for cDNA synthesis reactions using the ProtoScript^®^ II First Strand cDNA Synthesis Kit (BioLabs). The primers for *LEA13*, *LEA30*, *CaLEA73*, and *AtUBC10* were designed with Primer Express v.2.0 (Applied Biosystems) (Table S1). 

The absence of nonspecific products and primer dimers was confirmed by analyzing the amplicons’ melt curves and electrophoretic gel analysis. The cDNA templates and primers were added to SYBR GREEN/ROX qPCR PCR Master Mix (Thermo Scientific). qRT-PCR was performed with three biological and technical replicates using an Eco™ Illumina Real-Time PCR System, and the data were analyzed using the corresponding EcoStudy Software v5.0 program and normalized using the *AtUBC10* gene as internal control. All data were analyzed using Student’s *t*-test.

### 4.5. Analysis of Stomatal Development

Epidermal phenotypes were also determined on tissue cleared in 6% sodium hypochlorite for 2 h and stained with toluidine blue for 15 min. Images were captured from cotyledons of seedlings at 15 days post-germination (dpg) with a Leica DM5000 microscope. Differences between means were compared using the Student’s *t*-test (*p* < 0.05).

### 4.6. Transpiration and Stomatal Conductance

The transpiration and stomatal conductance were calculated from primary sensor readings of environmental factors, including temperature, relative humidity (RH), atmospheric pressure, and volumetric airflow, using the model 6400-15 Arabidopsis camera adapted to the IRGA LI-6400/XT equipment was used. The diameter of the chamber is 1 cm, and therefore, the area it covers is 0.74 cm^2^, the leaves used for the measurement were intended to cover an area of approximately 0.67 cm^2^, this camera is designed with transparent windows on the top and bottom of the sheet-covered Propafilm ^®^ film for use in natural light, all measurements were made in similar light conditions. An experiment setup was installed to measure the transpiration rate, photosynthesis, and stomatal conductance of 10 seedlings with 15 days post-germination (dpg) from *lea13*, *lea30*, and wild-type (WT). Differences between means were compared using the Student’s *t*-test (*p* < 0.05).

### 4.7. Statistical Analysis

Statistical analysis was conducted using GraphPad Prism 8.0.1 software. 16 plants were analyzed to evaluate drought, stomatal density, photosynthesis, transpiration, and stomatal conductance. Differences between means were compared using the Student’s *t*-test (*p* < 0.05).

### 4.8. Microarray Analysis in lea13 Genetic Background

The microarray procedure was performed by the Microarray Unit of the Institute of Cellular Physiology of the UNAM (http://microarrays.ifc.unam.mx/). This institute has a database of 29,950 genes that make up the complete genome of *A. thaliana*, which allows simultaneous analysis of many genes, yielding quantitative and reproducible data. The analyses were performed in triplicate from RNA extracted from 15 days old cotyledons. The *Col-0* ecotype was used as control, and the *lea13* line was considered as the variable. The selection of differentially expressed genes was performed with the software genArise (http://www.ifc.unam.mx/genarise/) by calculating the intensity-dependent Z-score. Differentially expressed the NCBI database obtained gene sequences, and the biological processes, molecular functions, and cellular components of upregulated and downregulated genes were examined using the Gene Ontology (GO) database. Functional gene annotation was analyzed with the DAVID tool (http://david.abcc.ncifcrf.gov/). Expression array data are accessible in NCBI’s Gene Expression Omnibus with the accession number GSE160866.

## Figures and Tables

**Table 1:** Up-regulated genes involved in hormones, stomatal development abiotic stress responses.

AGI Code	Zscore	Annotation	Description
AT1G65440	5.243761	GLOBAL TRANSCRIPTION FACTOR GROUP B, global transcription factor group B1	Transcription elongation factor SPT6-like protein
At1g75590	4.204775	F10A5.20, F10A5_20	SAUR-like auxin-responsive protein
At1g17240	4.004409	AtRLP2, F20D23.6, F20D23_6, receptor-like protein 2	Receptor-like protein 2; Encodes a CLAVATA2 (CLV2)-related gene.
At4g15040	3.362095	At4g15040	Subtilisin-like serine endopeptidase family protein.
At2g01420	3.242944	ARABIDOPSIS PIN-FORMED 4, AUXIN TRANSPORTER SPLICE VARIANT B, PIN-FORMED 4	Auxin efflux carrier component 4.
At3g05840	3.101374	ASKGAMMA, F10A16.14, F10A16_14	Shaggy-related protein kinase gamma; encodes a SHAGGY-like kinase involved in meristem organization.
At4g23270	3.003471	F21P8.160, F21P8_160, cysteine-rich RLK (RECEPTOR-like protein kinase) 19	Cysteine-rich receptor-like protein kinase 19
At3g62670	2.782405	ARR20, MEE41, maternal effect embryo arrest 41, response regulator ARR20	Putative two-component response regulator ARR20; response regulator 20
At1g76520	2.556337	F14G6.12, F14G6_12	Auxin efflux carrier family protein.
At1g65480	2.536566	F5I14.3, F5I14_3, FLOWERING LOCUS T	FLOWERING LOCUS T protein;
At2g39540	2.342906	F12L6.20, F12L6_20	Gibberellin-regulated protein
At5g10310	2.315854	F18D22.80, F18D22_80	Epidermal patterning factor-like protein

**Table 2:** Down-regulated genes involved in hormones, stomatal development abiotic stress responses.

AGI Code	Zscore	Annotation	Description
AT4G35580	−6.65111	F8D20.90, F8D20_90, NAC transcription factor-like 9	NAC transcription factor-like 9.
At5g65510	−4.88908	AT5G65510, AINTEGUMENTA-like 7, PLETHORA 7, PLT7	AINTEGUMENTA-like 7 protein; Encodes one of three PLETHORA transcription factors required to maintain high levels of PIN1 expression.
At3g61900	−4.14811	AT3G61900	SAUR-like auxin-responsive protein.
At2g06020	−3.48404	At2g06020	Myb family transcription factor; Homeodomain-like superfamily protein.
At5g26950	−3.45455	AT5G26950, AGAMOUS-like 93, F2P16.17, F2P16_17	Agamous-like MADS-box protein AGL93.
At3g20640	−3.19807	AT3G20640	Transcription factor bHLH123.
At5g40330	−3.18796	transcription factor MYB23	MYB DOMAIN PROTEIN 23, myb domain protein 23; Encodes a MYB gene that, when overexpressed ectopically, can induce ectopic trichome formation.
At1g26945	−3.06999	AT1G26945, KIDARI	Basic helix-loop-helix protein KIDARI; basic helix-loop-helix (bHLH) DNA-binding superfamily protein KIDARI (KDR).
At3g18040	−3.05605	AT3G18040, MAP kinase 9	Mitogen-activated protein kinase 9.
At1g01260	−3.02189	AT1G01260, F6F3.7, F6F3_7	Transcription factor bHLH13.
At3g25905	−2.89363	CLAVATA3/ESR-RELATED 27, CLE27	Protein CLAVATA3/ESR-related 27; Member of a large family of putative ligands homologous to the Clavata3 gene.
At5g58890	−2.82425	AGAMOUS-like 82, K19M22.9, K19M22_9	Protein AGAMOUS-LIKE 82.

## Data Availability

The original contributions generated for this study are included in the article and supplementary material. Expression array data are accessible in NCBI’s Gene Expression Omnibus with the accession number GSE160866.

## Supplementary Materials

The following are available online at https://www.mdpi.com/article/10.3390/plants10081694/s1, Figure S1. The average number of the cis-elements. ABRELATERD1 (ACGTG), DRECRTCOREAT (G/ACCGAC), MYBCORE (TAACTG), LTRE1HVBLT49 (CCGAC), and others in Cis elements in the promoter region of *Arabidopsis thaliana* LEA genes. The promoter regions were analyzed in the 0.5 kb upstream promoter region of the translation start site using the PLACE database. Figure S2. Schematic drawing of the average number of the cis-elements in the promoter region of *Arabidopsis thaliana* LEA genes. The promoter region was analyzed in the 0.5 kb upstream promoter region of the translation start site using the PLACE database. Exons are indicated by black boxes and the location of T-DNA insertions by gray triangles. The various regulatory boxes are shown in different colors. +1 indicates transcript start. Figure S3. Water Content (WC, %) in rosettes leaves of wild-type, *lea13*, and *lea30* plants. Water content was measured in 10 plants of the wild-type (ecotype Columbia-0), *lea13* and *lea30* lines after leaving them without watering for 15 days. *lea13* and *lea30* showed higher water loss compared to the wild-type plants at 10 and 15 days after cessation of irrigation. * Indicates a statistically significant difference at a 95% confidence level. Figure S4. In silico analysis of *LEA13* and *LEA30* expression in different plant tissues and stress conditions. Expression of *LEA13* and *LEA30* based on 20,000 RNA-seq libraries. Expression is measured in FPKM. Figure S5. LEA genes expression on drought conditions in the mutant background. (a) LEA13 expression was analyzed in plants of the *lea13* mutant background under drought conditions compared to normal irrigation conditions. LEA13 expression increased under drought conditions in all three tissues analyzed. (b) The same observations were made for *LEA30*; its expression decreased. In both cases, the increase was more evident in inflorescences. * Indicates a statistically significant difference between control and water deficit conditions at a 95% confidence level. Figure S6. Expression analysis of LEA genes in different genetic backgrounds. To find out whether the expression of LEA genes can be affected by the expression of another LEA gene. We analyzed the expression of *LEA13* in the lea30 background and vice versa. The results showed (a) LEA13 increased its expression in the *lea30* background in leaf and stem, but not in inflorescences. (b) *LEA30* did not increase its expression concerning wild-type background. * Indicates a statistically significant difference between control and water deficit conditions at a 95% confidence level. Table S1. Primers sequences used in this study.
