# Supplementary figures and images for: LEA13 and LEA30 Are Involved in Tolerance to Water Stress and Stomata Density in Arabidopsis thaliana
Source: Plants (Basel). 2021 Aug 18;10(8):1694. doi: 10.3390/plants10081694 (PMC8400336; doi:10.3390/plants10081694)

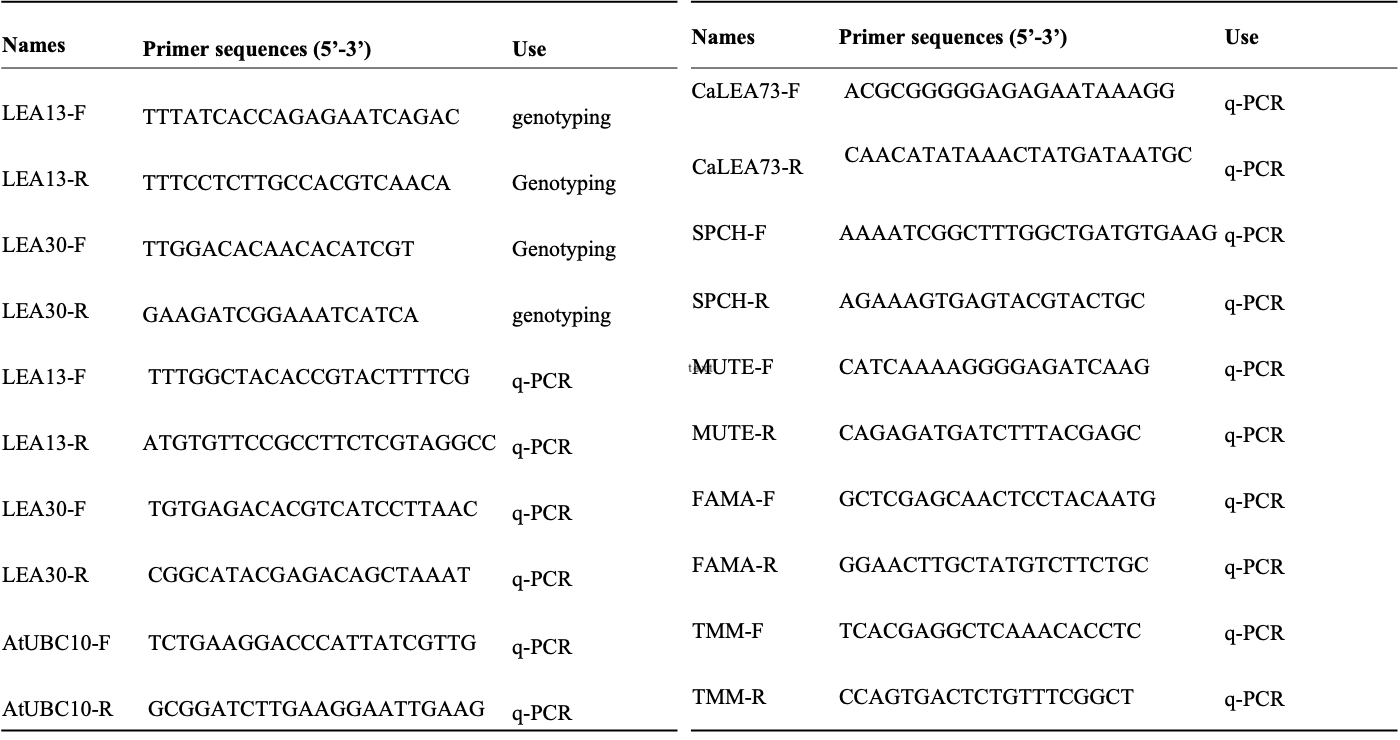

Supplement: Supplementary file 1 [file plants-10-01694-s001.zip › Supplementary material LopezCordova A/Table S1 Lopez-Cordova A Plants.tiff]

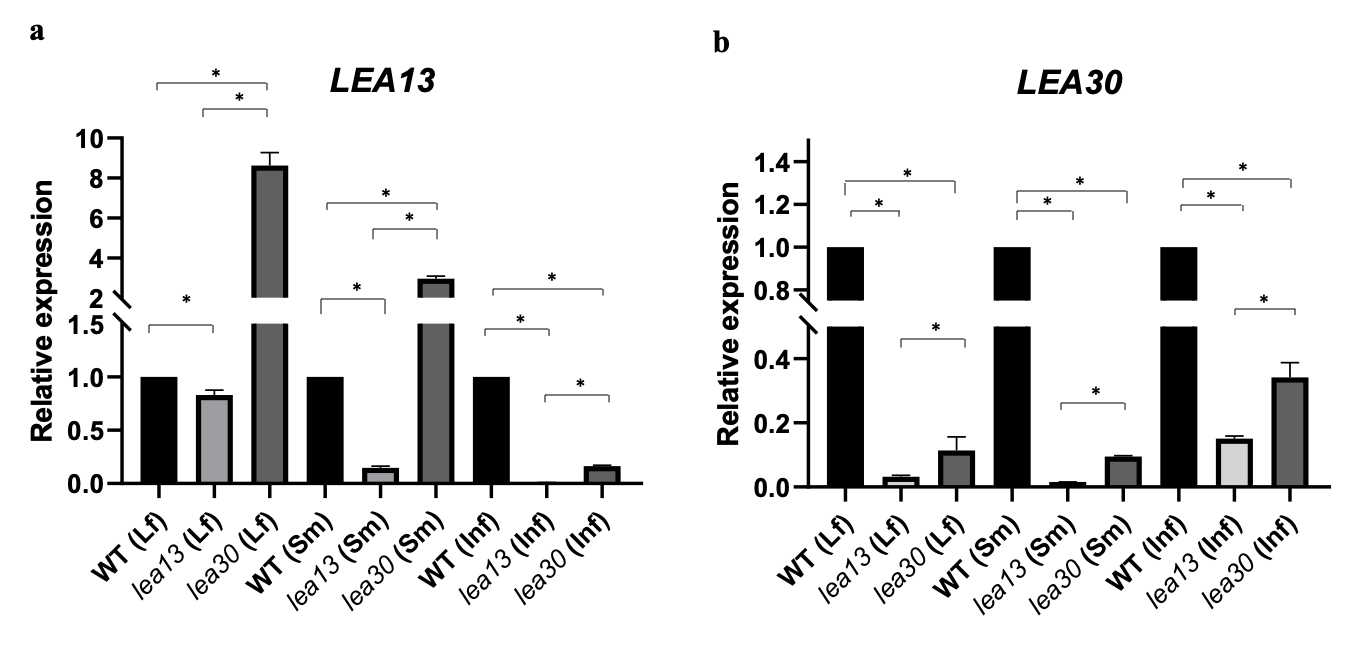

Supplement: Supplementary file 1 [file plants-10-01694-s001.zip › Supplementary material LopezCordova A/Figure S6 Lopez-Cordova A Plants.tiff]

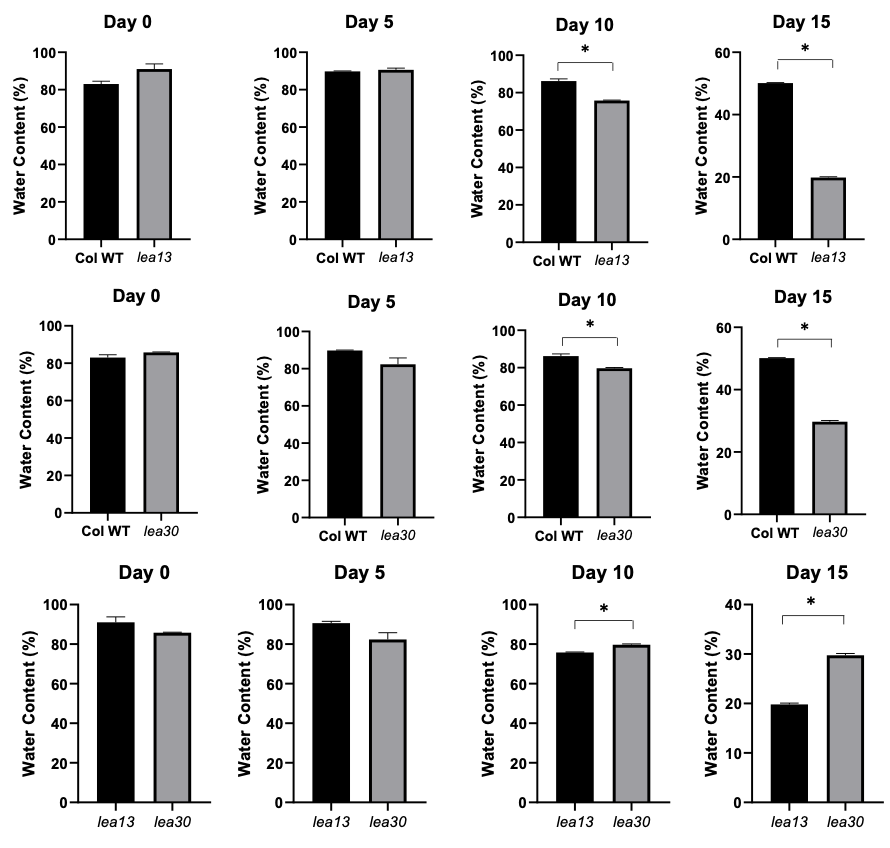

Supplement: Supplementary file 1 [file plants-10-01694-s001.zip › Supplementary material LopezCordova A/Figure S3 Lopez-Cordova A Plants.tiff]

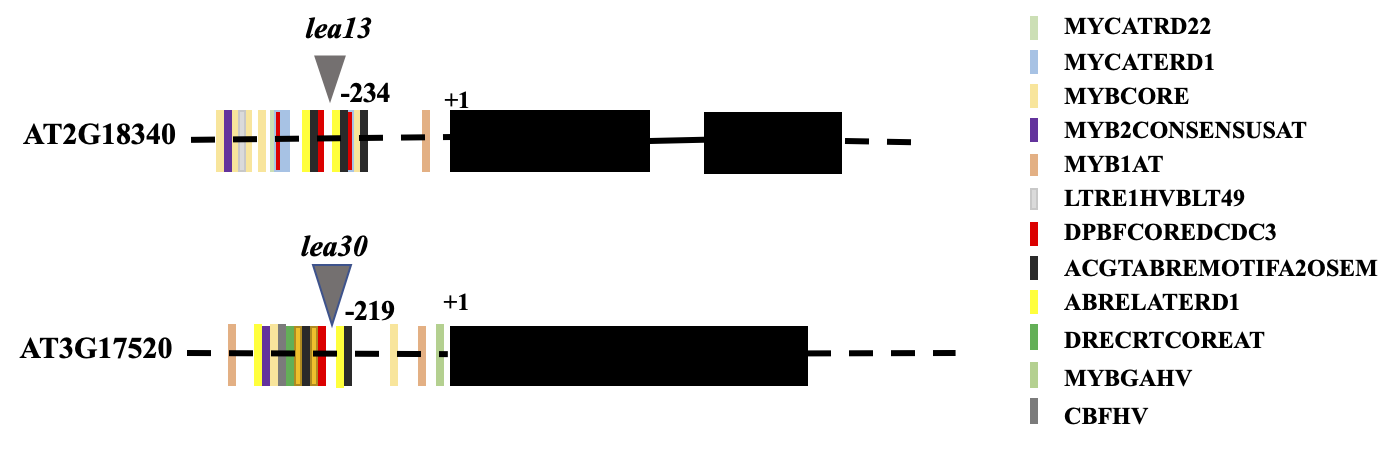

Supplement: Supplementary file 1 [file plants-10-01694-s001.zip › Supplementary material LopezCordova A/Figure S2 Lopez Cordova A Plants.tiff]

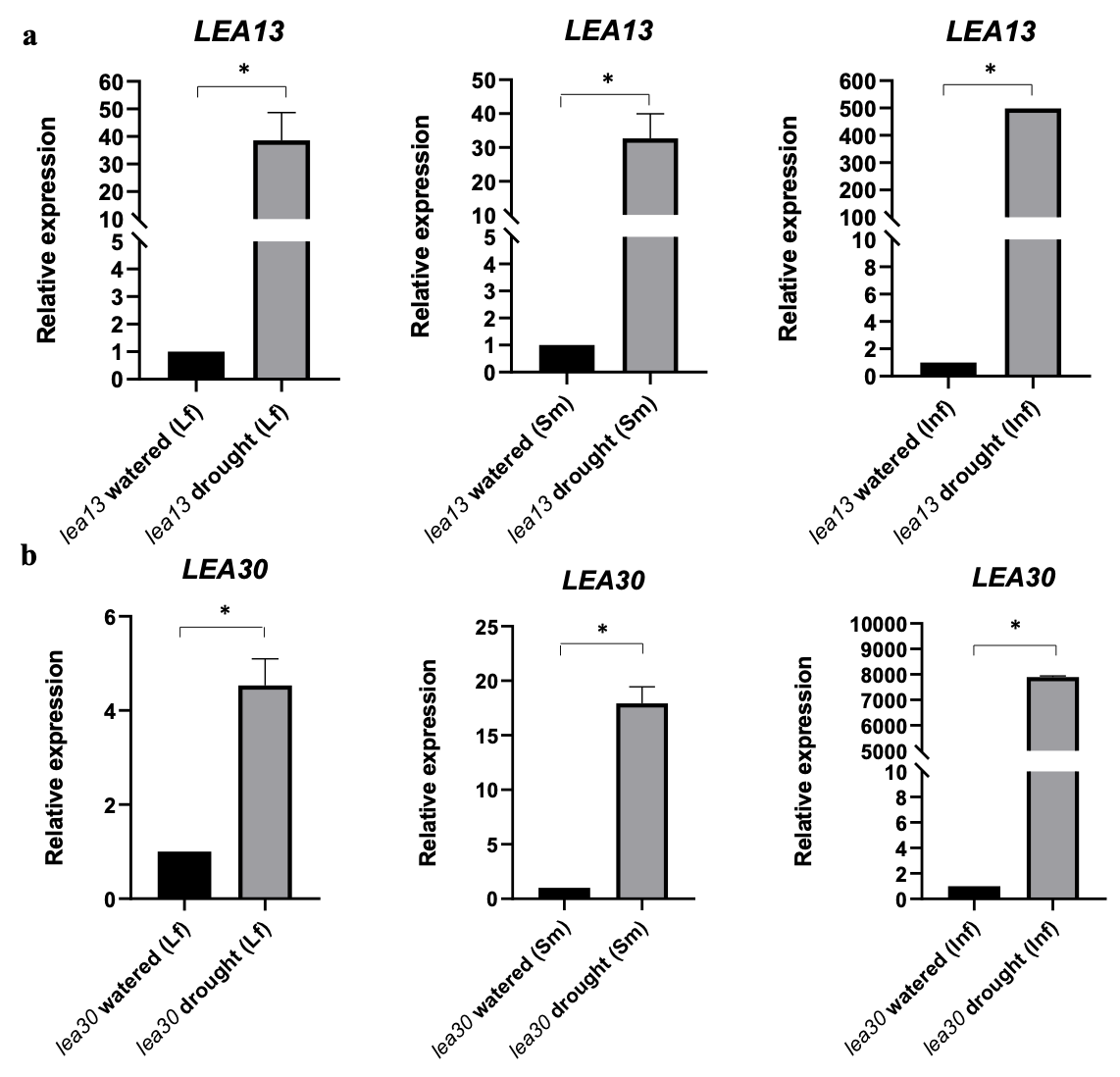

Supplement: Supplementary file 1 [file plants-10-01694-s001.zip › Supplementary material LopezCordova A/Figure S5 Lopez-Cordova A Plants.tiff]

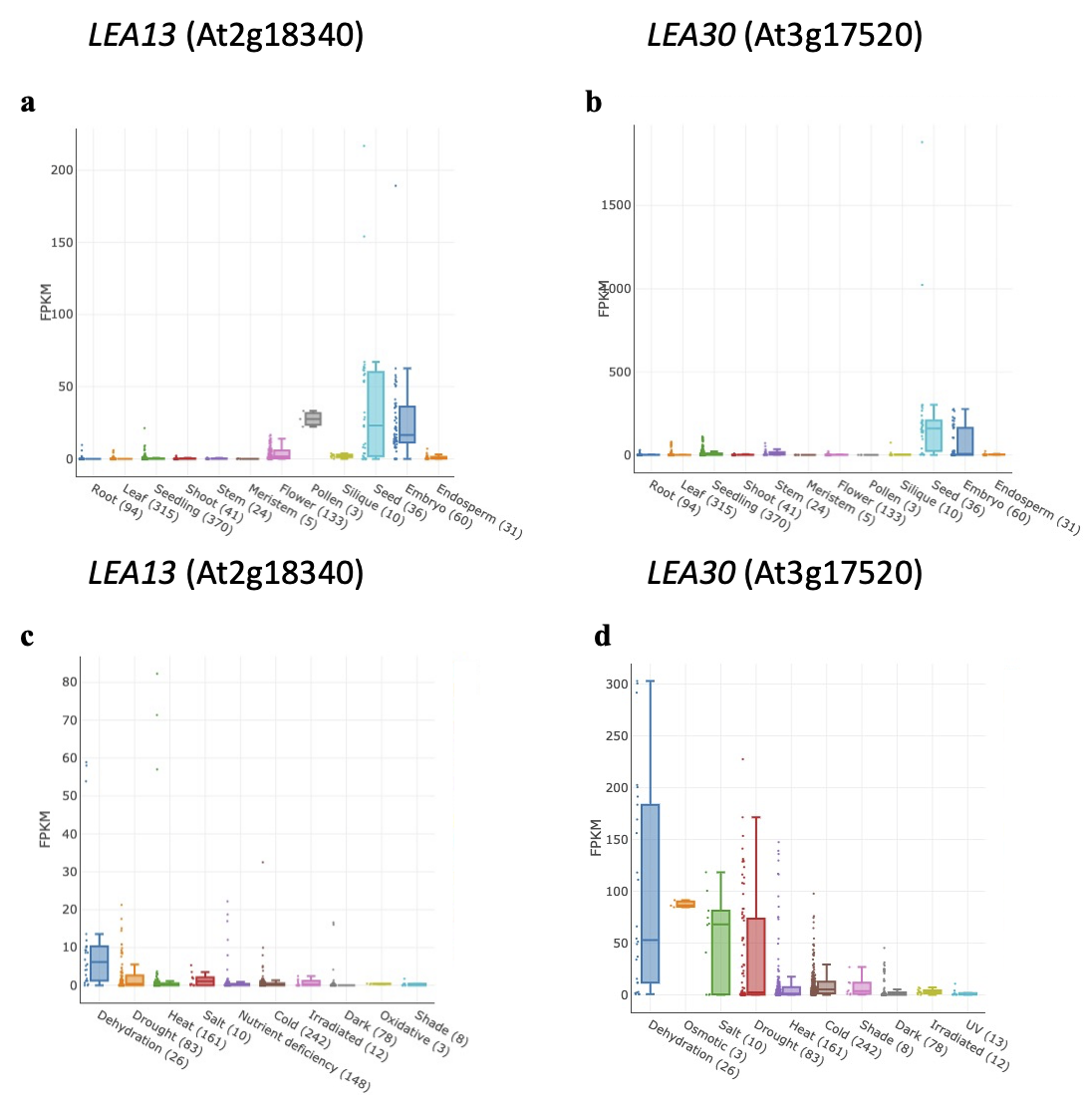

Supplement: Supplementary file 1 [file plants-10-01694-s001.zip › Supplementary material LopezCordova A/Figure S4 Lopez-Cordova A Plants.tiff]

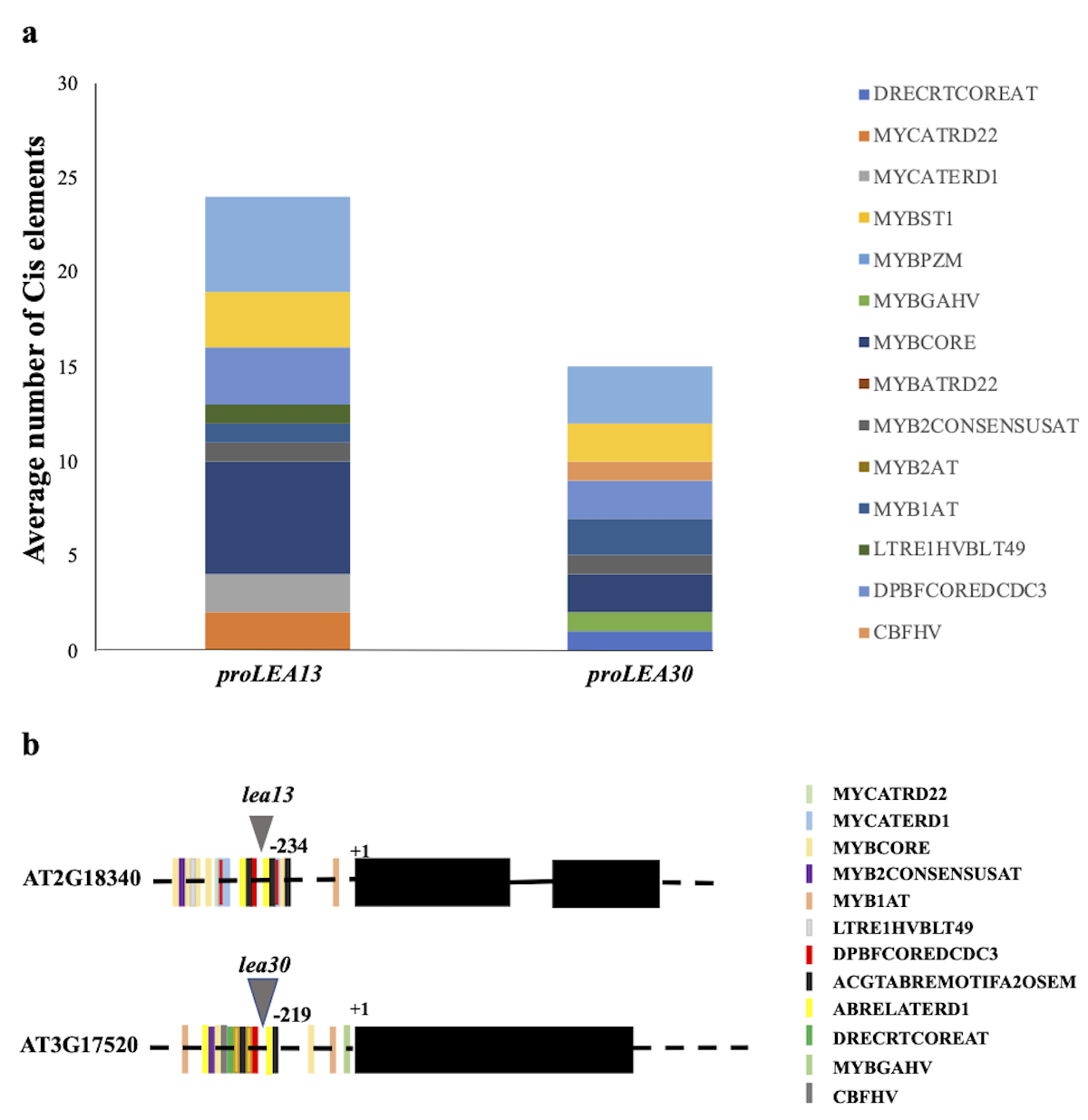

Supplement: Supplementary file 1 [file plants-10-01694-s001.zip › Supplementary material LopezCordova A/Figure S1 Lopez-Cordova A Plants.tiff]
